# Generative AI acceptance in study-travel contexts: an integrative model of affective and utilitarian pathways with mediation and grade-level moderation

**DOI:** 10.3389/fpsyg.2026.1961221

**Published:** 2026-10-06

**Authors:** Yuan Li, Dexin Li

**Affiliations:** 1 School of Management, Chongqing University of Science and Technology, Chongqing, China; 2 School of Architecture, Tianjin University, Tianjin, China

**Keywords:** adoption intention, generative AI, grade moderation, perceived enjoyment, perceived risk, study-travel, UTAUT2

## Abstract

**Background:**

Generative artificial intelligence is progressively reshaping study-travel—a curriculum-embedded educational practice. Yet, the psychological processes governing upper-primary and middle-school students’ adoption of such technology in this context remain underexplored. Prior technology acceptance inquiries have predominantly centered on adult populations, leaving adolescent cognitive-developmental contingencies unaddressed.

**Objective:**

This investigation aimed to elucidate the differential roles of affective versus utilitarian drivers in shaping study-travel participants’ anticipated intentions to adopt GenAI, and to determine whether these pathways differ by grade level.

**Method:**

We constructed an integrative model grounded in the extended Unified Theory of Acceptance and Use of Technology (UTAUT2), supplemented by Piaget’s developmental framework. The model incorporated perceived enjoyment as a mediator and perceived risk as a direct negative antecedent, with grade level specified as a moderator of selected pathways. Survey data from 741 students in grades 4–9, drawn from twelve schools across six Chinese cities, were analyzed using structural equation modeling, multi-group comparisons, and cluster-robust standard errors to account for the nested sampling structure. Measurement invariance across grade groups was established prior to structural comparisons.

**Results:**

Perceived enjoyment emerged as the largest estimated predictor of adoption intention in this sample (*β* = 0.43, *p* < 0.001), surpassing performance expectancy (*β* = 0.30, *p* < 0.001). Enjoyment fully mediated social influence effects and partially transmitted performance expectancy‘s influence. Perceived risk exerted a significant suppressive effect (*β* = −0.24, *p* < 0.001), with primary students exhibiting approximately twice the inhibitory magnitude of middle-school counterparts. Grade significantly moderated the performance expectancy–intention relationship, and provided borderline evidence for moderation of the risk–intention relationship, indicating systematic grade-related differences in how these pathways operate.

**Conclusion:**

These findings advance technology acceptance theory by introducing grade-related contingency and establishing an “affective–efficacy” dual-channel model. Practical implications for designing grade-differentiated educational AI products are discussed.

## 1 Introduction

Study-travel—defined as curriculum-anchored off-campus inquiry integrated with travel—has become deeply embedded in China‘s basic education system. By 2024, the study-travel market reached approximately 179 billion RMB, reflecting sustained growth in both supply and demand (iiMedia Research, 2024). Such scale generates substantial opportunities for technological augmentation.

Parallel to this expansion, generative artificial intelligence (GenAI) is rapidly infiltrating educational spaces. Its conversational, multimodal capabilities could fundamentally alter study-travel pedagogy: personalizing pre-trip preparation, furnishing real-time cultural interpretation on-site, and scaffolding post-trip synthesis. These affordances promise to disrupt the longstanding pattern of superficial information acquisition and low-order knowledge reproduction that has limited study-travel‘s educational potential (Chen and Zheng, 2026; Li et al., 2025).

*Yet a critical question persists*: what psychological conditions facilitate or impede students’ willingness to embrace GenAI as a cognitive companion during study-travel? This question is non-trivial because adolescent technology adoption involves developmental, affective, and contextual factors that diverge substantially from adult populations (Zhou and Wang, 2026; Lin and Hu, 2026).

Extant technology acceptance research manifests two notable lacunae when projected onto this context. *First, a population gap*. The vast majority of studies have sampled university students or adult professionals. Even when adolescents appear, they are often collapsed into a homogeneous “digital native” category—an aggregation that obscures the cognitive transformations unfolding across later childhood and early adolescence (grades 4–6 to grades 7–9), a period during which information evaluation, risk calibration, and decision-making processes undergo substantial reorganization (Piaget, 1950; Inhelder and Piaget, 1958; Crone and Dahl, 2012). *Second, a contextual gap*. Generic learning settings differ qualitatively from study-travel, which is distinguished by situational immediacy (knowledge needs arise spontaneously from the travel milieu), temporal transience (information demands are acute and fleeting), and hedonic immersion (enjoyment is integral to the experience). These distinctive features suggest that the psychological drivers of technology adoption may tilt from instrumental value optimization toward experiential enjoyment maximization—a proposition consistent with the rising emphasis on hedonic motivations in consumer technology research (Venkatesh et al., 2012; Lin and Hu, 2026). While recent scholarship has begun to explore AI‘s hedonic functions in tourism, such work has not systematically addressed adolescent populations nor interrogated how risk perception operates at the intersection of developmental stage and travel-learning context (Chen and Zheng, 2026).

The present investigation aims to bridge these intersecting gaps. We anchor our inquiry in UTAUT2 (Venkatesh et al., 2012) yet undertake a theoretically motivated structural adaptation calibrated to study-travel’s unique ecology and the cognitive characteristics of upper-primary and middle-school students. Specifically, we introduce perceived enjoyment as a mediator and perceived risk as a direct negative antecedent, designate grade level as a moderator of key pathways, and formulate two interlocking research questions: (1) Which psychological drivers and inhibitors most powerfully shape students’ GenAI adoption intentions in study-travel, and what are their relative magnitudes? (2) Do these pathways exhibit systematic differences as a function of grade level?

## 2 Literature review and theoretical framework

### 2.1 Adapting UTAUT2 to the study-travel setting

The Unified Theory of Acceptance and Use of Technology (UTAUT), introduced by Venkatesh et al. (2003), integrated eight prior technology acceptance models into a parsimonious framework centered on performance expectancy, effort expectancy, social influence, and facilitating conditions. Recognizing the limitations of these workplace-oriented constructs for explaining consumer technology adoption, Venkatesh et al. (2012) extended the model to UTAUT2, incorporating hedonic motivation, price value, and habit—variables more germane to voluntary, consumption-driven use contexts. This extended framework has since been widely deployed to investigate adoption of mobile services, social media, and AI-enabled applications (Dwivedi et al., 2019; Tamilmani et al., 2021).

When transplanting UTAUT2 to the study-travel domain, we undertook deliberate contextual re-specification. First, GenAI use in study-travel is student-initiated and non-obligatory, carrying substantial hedonic valence—making hedonic motivation directly relevant. Second, because students incur no personal financial cost, price value was excluded. Third, our sample predominantly involved first-time or early-stage users, rendering habit‘s influence minimal (Gupta et al., 2022). We consequently retained performance expectancy, effort expectancy, and social influence, while operationalizing hedonic motivation as perceived enjoyment—a construct capturing the intrinsic pleasure derived from GenAI interaction during study-travel.

*Performance expectancy*—the conviction that technology enhances task outcomes—was contextually reframed as students’ perceptions that GenAI expedites destination knowledge acquisition, deepens cultural-historical understanding, and facilitates inquiry planning. Given that middle-school study-travel increasingly features project-based and cross-disciplinary components, we anticipated that performance expectancy‘s predictive weight would be stronger among middle-school students, reflecting their greater capacity to link current tool-use to distal learning goals (Inhelder and Piaget, 1958).

*Effort expectancy*—perceived ease of use—acquires particular salience for students in grades 4–9, whose operational proficiencies remain emergent. GenAI’s conversational interface, supplanting traditional menu hierarchies with natural dialogue, substantially lowers interaction barriers. When students discover they can obtain responsive, meaningful answers through intuitive chat, self-efficacy and exploratory motivation are enhanced (Scherer et al., 2019).

*Social influence*—the perceived expectations or encouragement from valued referents—assumes a dual “authority–peer” configuration among adolescents. Teachers function as both knowledge authorities and technology-use norm-setters. Peers, through intensive interaction during shared study-travel experiences, generate immediate modeling and conformity pressures. Parents, though not physically present, shape judgments through everyday conversations about technology and learning. We posited that these tripartite social forces operate primarily by cultivating an atmosphere of collective engagement (“everyone is using it, and it is enjoyable”) rather than imposing direct normative compliance—a pathway implicating affective contagion more than subjective norm internalization (Lin and Hu, 2026).

### 2.2 Perceived enjoyment as the affective hub

Study-travel is not a classroom transported to a different venue. Its inherent touristic character means participants approach the activity with elevated expectations of novelty, entertainment, and discovery (Wang et al., 2024). GenAI, by generating historical anecdotes, posing situated puzzles, crafting personalized narratives, and enabling character-based role-play, readily elicits curiosity and delight. Developmental research consistently demonstrates that late childhood and early adolescent decision-making is heavily swayed by affective valence (Crone and Dahl, 2012; Steinberg, 2005). In educational settings, emerging evidence indicates that perceived enjoyment predicts AI learning companion adoption more robustly than perceived usefulness (Huang et al., 2025; Zhou and Wang, 2026). In study-travel—where entertainment expectations are heightened—we hypothesized that perceived enjoyment would occupy the apex of the driver hierarchy and might function as a pathway through which instrumental perceptions are associated with behavioral intentions. Absent enjoyment, a potent functional capability may fail to translate into adoption.

### 2.3 Perceived risk: information anxiety and cognitive inertia

Drawing on a preliminary open-ended survey (*N* = 87 students) and relevant literature, we conceptualized risk concerns among study-travel participants along two core dimensions. Information risk encompasses worries that “AI might fabricate information” or “could misattribute historical facts.” This concern is particularly acute among primary students, whose logical reasoning capacities remain underdeveloped and who may lack the background knowledge required to detect inaccuracies (Schmidt and Cleveland, 2023). Cognitive dependency risk captures a more introspective concern—“over-reliance on AI may weaken my own thinking.” This reflects a nascent yet perceptive metacognitive alertness to technology‘s potential to erode personal cognitive agency. In contrast to privacy risk, which younger students may not yet prioritize due to limited privacy literacy (Livingstone and Stoilova, 2021), these two dimensions are developmentally sensitive and contextually grounded. We note that privacy risk was part of the initial item pool but was removed due to low factor loading, as detailed in the Methods section. We predicted that perceived risk would directly suppress adoption intention, and that this suppression would be more pronounced among primary students than among middle-school students.

### 2.4 Grade level as a moderator

Piaget’s epistemological framework, supplemented by contemporary developmental research on adolescent metacognition and decision-making (Crone and Dahl, 2012; Steinberg, 2005), supplies the developmental optic for this inquiry. Upper-primary students (grades 4–6) predominantly operate within the late concrete operational stage, reasoning about tangible objects and privileging surface-level experiential cues—visual personification, vocal emotionality, immediate feedback—when appraising technological artifacts (Piaget, 1950). Middle-school students (grades 7–9), in contrast, have typically entered the formal operational stage, characterized by abstract reasoning, hypothetical-deductive logic, and counterfactual thinking. They are better positioned to systematically evaluate information credibility, triangulate across sources, and devise contingent strategies (Inhelder and Piaget, 1958). This developmental contrast yields clear predictions: grade should positively moderate the performance expectancy–intention relationship (the instrumental pathway is stronger among middle-school students) and negatively moderate the perceived risk–intention relationship (risk‘s deterrent effect is weaker among middle-school students). For the enjoyment pathway, given that study-travel perennially supplies high hedonic expectations, we anticipated minimal grade differences. We note that grade level may reflect multiple factors including cognitive development, school context, and instruction, and we interpret observed differences accordingly.

### 2.5 Conceptual model

The theoretical arguments converge on a conceptual model wherein adoption intention (AI) serves as the ultimate dependent variable. Positive antecedents comprise performance expectancy (PE), effort expectancy (EE), social influence (SI), and perceived enjoyment (PEJ); the negative antecedent is perceived risk (PR). PEJ is additionally specified as a mediator between PE and AI. Grade level moderates the PE → AI and PR → AI pathways. Table 1 presents a complete summary of all hypothesized paths, directions, expected relative strengths, and theoretical rationales.

**Table 1:** Conceptual model: hypothesized paths and expected effects.

Path	Direction	Expected strength	Theoretical rationale
PE → AI	+	Moderate	Direct instrumental drive
PE → PEJ	+	Strong	Functional value translates into affective experience
PEJ → AI	+	Strongest	Affective primacy in hedonic contexts
EE → AI	+	Weak	Ease-of-use as a hygiene factor
SI → PEJ	+	Moderate	Peer/teacher endorsement enhances enjoyment
SI → AI	+ (Indirect only)	Non-significant direct	Fully mediated by PEJ
PR → AI	−	Moderate	Risk concerns suppress adoption
Grade moderates: PE → AI	+ (Positive)	−	Stronger among middle-school students
Grade moderates: PR → AI	− (Negative)	−	Weaker inhibition among middle-school students

All paths derived from UTAUT2 integrated with Piaget‘s cognitive developmental theory and contemporary developmental research. PE, performance expectancy; EE, effort expectancy; SI, social influence; PEJ, perceived enjoyment; PR, perceived risk; AI, adoption intention.

## 3 Methods

### 3.1 Instrument development

All latent variable items were developed through a systematic four-phase procedure: original scale acquisition → study-travel contextualization → child-appropriate language adaptation → cognitive validation. Item sources included Venkatesh et al. (2012) for UTAUT2 constructs and Featherman and Pavlou (2003) for perceived risk. The original English scales were translated into Chinese and back-translated by two independent bilingual researchers to ensure conceptual equivalence. Contextualization involved substituting “work” with “study-travel tasks” and “system” with “generative AI tool.” Cognitive interviews with eight students across grades 4, 6, and 8 guided revisions of approximately one-third of the items to ensure age-appropriate comprehension. For instance, the effort expectancy item “My interaction with the system is clear and understandable” was rephrased as “It is easy for me to chat with the AI and make it understand my meaning.”

The initial perceived risk scale comprised three items: PR1 (information risk: “I’m a bit worried that the study-travel knowledge AI provides might be wrong”), PR2 (cognitive dependency risk: “I’m afraid that if I use AI too much, my willingness to look up information and think for myself will decrease”), and PR3 (privacy risk: “I’m slightly concerned that my questions and personal information might be recorded and misused by AI”). An exploratory factor analysis on the three items revealed that PR3 loaded at *λ* = 0.48, substantially below the 0.55 threshold; it was therefore removed. The remaining two items showed a correlation of *r* = 0.58 and demonstrated acceptable internal consistency for a two-item composite (Spearman-Brown coefficient = 0.73). We opted to treat perceived risk as an observed composite (the mean of PR1 and PR2) rather than a two-indicator latent factor because a latent factor with only two indicators is just-identified and tends to produce unreliable estimates (Kline, 2016, as cited in the methodological literature).

The initial instrument comprised 23 items: 19 antecedent items across five constructs (PE 4, EE 4, SI 4, PEJ 4, PR 3) plus 4 adoption intention items. After removing PR3 due to a factor loading below 0.55, the final instrument comprised 22 items: 18 antecedent items (PE 4, EE 4, SI 4, PEJ 4, PR 2) plus 4 adoption intention items. The final model included five latent constructs (PE, EE, SI, PEJ, AI) and one observed composite variable (PR). The five latent constructs were measured by 20 indicators; PR was computed as the mean of PR1 and PR2. Complete item listings and descriptive statistics appear in Table 2.

**Table 2:** Measurement items with contextualized phrasing and descriptive statistics.

Construct	Code	Item	Mean	SD
Performance Expectancy (PE)	PE1	Using AI helps me understand the background of a study-travel site more quickly	3.96	0.88
	PE2	AI can help me organize the questions and steps I need for my research tasks	3.87	0.93
	PE3	When I encounter something I don‘t understand during the trip, AI gives helpful explanations	4.02	0.85
	PE4	Overall, AI helps me complete my study-travel tasks better	3.78	0.97
Effort Expectancy (EE)	EE1	Learning to chat with AI to help me with study-travel is very easy for me	4.15	0.79
	EE2	I can easily make AI understand what I mean	4.05	0.82
	EE3	I do not need to spend a long time to become skilled at using AI	4.20	0.76
	EE4	Even the first time I use AI, I find communication smooth	4.08	0.84
Social Influence (SI)	SI1	My teacher thinks using AI in study-travel is a good idea	3.65	1.02
	SI2	Many of my classmates use AI to help with their study-travel tasks	3.89	0.96
	SI3	My friends think using AI to assist study-travel is cool	3.74	1.01
	SI4	My family supports me using AI appropriately during study-travel	3.85	0.95
Perceived Enjoyment (PEJ)	PEJ1	Chatting with AI about the interesting stories and legends related to the study-travel site is enjoyable	4.12	0.85
	PEJ2	AI‘s ability to answer my questions in a storytelling way fascinates me	4.05	0.89
	PEJ3	Using AI to generate a short script or poem about the study-travel experience makes me happy	3.98	0.92
	PEJ4	Exploring all the features of AI during study-travel is itself a source of fun	4.09	0.87
Perceived Risk (PR)	PR1	I’m a bit worried that the study-travel knowledge AI provides might be wrong	3.02	1.05
	PR2	I‘m afraid that if I use AI too much, my willingness to look up information and think for myself will decrease	2.76	1.12
	PR3	I’m slightly concerned that my questions and personal information might be recorded and misused by AI	2.47	1.15
Adoption Intention (AI)	AI1	In the next study-travel trip, I‘m willing to let AI be my learning partner	4.15	0.87
	AI2	I would recommend to my classmates that they use AI to enrich their study-travel experience	3.96	0.92
	AI3	If conditions permit, I would use AI frequently during study-travel	4.02	0.89
	AI4	I plan to try AI tools in the upcoming study-travel trip	4.08	0.91

PR3 was removed due to factor loading < 0.55; final perceived risk measured with PR1 and PR2 as a composite variable (mean of the two items).

### 3.2 Sample and data collection

A stratified convenience sampling strategy was implemented following a “region–city–school–class” nested design. Data collection occurred from September to October 2025 across six cities representing China‘s eastern (Nanjing, Guangzhou), central (Wuhan, Zhengzhou), and western (Chengdu, Xi’an) regions. In each city, one primary and one middle school were recruited, encompassing grades 4 through 9. Research assistants administered surveys in classroom settings under standardized protocols, using both paper and QR-code formats. Of the 892 distributed questionnaires, 641 (71.9%) were administered via paper format and 251 (28.1%) via QR-code format. For paper questionnaires, completion time was recorded by research assistants using stopwatches, with the start time recorded at the moment the survey was distributed and the end time at the moment of collection. Parental written informed consent was obtained prior to participation, and students provided verbal assent. Of 892 distributed questionnaires, 741 valid responses were retained after insufficient completion time on paper questionnaires (<120 seconds; *n* = 48), failed attention-check items (*n* = 62), or patterned responding (*n* = 41). The final sample comprised 348 primary students (grades 4–6, 46.96%) and 393 middle-school students (grades 7–9, 53.04%); 365 male, 376 female; 58.7% had three or more prior study-travel experiences; 11.9% used GenAI weekly, 52.8% occasionally, and 35.3% had heard of but not used it. These proportions broadly align with national participation patterns. Participants were asked to respond based on their general exposure to and knowledge of generative AI tools (e.g., ChatGPT, DeepSeek, or similar conversational AI systems), as well as their anticipated use in hypothetical study-travel scenarios. No specific AI tool or live demonstration was provided during the survey; thus, responses for non-users reflect anticipated rather than experienced perceptions (see Table 3).

**Table 3:** Measurement invariance tests across grade groups.

Model	*χ* ^2^	df	CFI	TLI	RMSEA	SRMR	ΔCFI
Configural	487.23	198	0.951	0.943	0.048	0.041	—
Metric	498.51	207	0.943	0.938	0.049	0.045	0.008
Scalar	519.87	216	0.932	0.927	0.052	0.048	0.011
Partial scalar	508.12	215	0.940	0.935	0.050	0.046	0.008

ΔCFI is calculated relative to the previous model. The partial scalar model frees the intercept for SI1 (social influence item 1) based on modification indices. ΔCFI ≤ 0.01 indicates invariance. Metric invariance is supported; partial scalar invariance is used for latent mean comparisons.

### 3.3 Analytical procedures

Preliminary data processing and common method bias diagnostics were performed using SPSS 26.0. Structural equation modeling (SEM) was conducted in Mplus 8.7 using MLR estimation (maximum likelihood with robust standard errors). Likert indicators were treated as continuous, consistent with SEM conventions for five or more response categories. Missing data were handled with FIML. Perceived risk was specified as an observed composite variable calculated as the mean of PR1 and PR2, rather than as a latent factor, due to the limited reliability of a two-indicator latent construct. To account for the nested sampling structure (students clustered within schools), we re-estimated the model using cluster-robust standard errors (TYPE = COMPLEX) with school as the clustering unit. Multi-group analysis comparing primary and middle-school groups was employed for moderation testing, with ΔCFI ≥ 0.01 as the primary criterion and significant Δ*χ*^2^ as a supporting indicator. All *χ*^2^ difference tests were performed using the Satorra–Bentler scaled chi-square difference test, which is the appropriate correction for MLR estimation. Prior to structural comparisons, we conducted multi-group confirmatory factor analysis (MG-CFA) to establish measurement invariance across grade groups, testing configural, metric, and scalar invariance with ΔCFI ≤ 0.01 as the criterion for invariance.

Bias-corrected bootstrapped mediation tests (5,000 resamples) were used to test indirect effects.

## 4 Results

### 4.1 Common method bias and measurement properties

Harman‘s single-factor test on unrotated exploratory factor analysis extracted seven factors with eigenvalues exceeding 1.0, with the first factor accounting for 27.33% of total variance—substantially below the 40% threshold, suggesting that common method bias is not a predominant concern. To further assess this issue, we supplemented the Harman test with an unmeasured latent method factor (ULMC) approach. The ULMC model included all indicators loading on both their respective trait factors and a single common method factor. The method factor was specified with all factor loadings constrained equal to ensure identification (Podsakoff et al., 2003). The method factor accounted for 9.8% of the total variance (calculated as the squared multiple correlation from the method factor loadings), below the 10% recommended threshold (Williams et al., 1989), further supporting the conclusion that common method variance does not substantially threaten the validity of our findings.

The CFA measurement model was re-estimated after re-specifying perceived risk as an observed composite variable. The model was estimated with all latent constructs (PE, EE, SI, PEJ, AI) specified as reflective latent factors, each with 3–4 indicators. PR was entered as a single observed composite indicator (the mean of PR1 and PR2). The measurement invariance testing proceeded as follows. The configural model included 20 indicators loading on five latent constructs. In the metric invariance model, factor loadings were constrained equal across groups. However, due to the limited number of indicators per construct and the small number of groups (2), we applied partial metric invariance constraints, releasing loadings that showed non-negligible cross-group differences based on modification indices. Specifically, two loadings (PE2 and SI3) were freed across groups, as reflected by the df increase in Table 3. For the scalar model, we similarly applied partial scalar invariance, releasing one intercept (SI1), which yielded the additional df increase. This approach follows the recommendations of Byrne et al. (1989) and Chen (2007) for partial invariance in multi-group CFA. The final re-estimated model exhibited acceptable fit: *χ*^2^ = 512.47, df = 209, *χ*^2^/df = 2.45, RMSEA = 0.044, CFI = 0.962, TLI = 0.954, SRMR = 0.039. The fit indices remain identical to the original specification because the composite indicator is treated as an observed variable with no estimated parameters (no factor loading, no residual variance); consequently, the degrees of freedom and chi-square are unchanged. Standardized factor loadings for the latent constructs ranged from 0.69 to 0.90, all statistically significant at *p* < 0.001. Composite reliability (CR) values ranged from 0.82 to 0.93, exceeding the recommended minimum of 0.70; average variance extracted (AVE) ranged from 0.55 to 0.75, all surpassing 0.50, supporting convergent validity. For discriminant validity, we calculated HTMT values, all of which were below 0.75 (below the 0.85 threshold), in addition to confirming that all AVE square roots exceeded corresponding inter-construct correlations. Perceived risk (PR) was treated as an observed composite variable (mean of PR1 and PR2) in all analyses; therefore, CR, AVE, and √AVE are not reported for this construct.

We also tested measurement invariance for the perceived risk composite across grade groups using a two-group confirmatory factor model with PR1 and PR2 as two indicators (with loadings constrained for identification). The model showed acceptable configural fit and metric invariance was supported (ΔCFI = 0.007), suggesting that the risk items were understood comparably across primary and middle-school students.

Means, standard deviations, and the discriminant validity matrix are presented in Table 4.

**Table 4:** Descriptive statistics and discriminant validity matrix.

Variable	Mean	SD	CR	AVE	1	2	3	4	5	6
1. PE	3.91	0.82	0.90	0.69	**0.83**					
2. EE	4.12	0.78	0.88	0.65	0.49	**0.81**				
3. SI	3.78	0.88	0.87	0.63	0.52	0.45	**0.79**			
4. PEJ	4.06	0.84	0.93	0.75	0.55	0.44	0.51	**0.87**		
5. PR	2.89	0.95	—	—	−0.30	−0.18	−0.16	−0.33	**—**	
6. AI	4.05	0.85	0.92	0.74	0.53	0.41	0.47	0.64	−0.40	**0.86**

PR was treated as an observed composite variable (mean of PR1 and PR2); therefore CR, AVE, and √AVE are not applicable. Diagonal bold values are square roots of AVE for latent constructs. HTMT values all below 0.75, supporting discriminant validity. PE, performance expectancy; EE, effort expectancy; SI, social influence; PEJ, perceived enjoyment; PR, perceived risk; AI, adoption intention.

Prior to structural comparisons across grade groups, we established measurement invariance. The configural model showed acceptable fit. The metric invariance model yielded ΔCFI = 0.008 from the configural model, supporting metric invariance. The scalar invariance model yielded ΔCFI = 0.011 from the metric model; given that this slightly exceeded the 0.01 criterion, we used partial scalar invariance with one intercept freed (SI1). These results justify cross-group structural comparisons.

### 4.2 Direct effects and mediation

The structural model demonstrated acceptable fit: *χ*^2^/df = 2.61, RMSEA = 0.047, CFI = 0.957, TLI = 0.950, SRMR = 0.045. Path coefficients (Table 5) revealed that perceived enjoyment had the largest estimated standardized coefficient in this model (*β* = 0.432, *p* < 0.001), surpassing performance expectancy (*β* = 0.301, *p* < 0.001). To formally compare these coefficients, we conducted a Wald test of coefficient equality using cluster-robust standard errors. The difference between PEJ (*β* = 0.425) and PE (*β* = 0.298) was statistically significant (*χ*^2^ = 4.12, *p* = 0.042), supporting the descriptive observation that perceived enjoyment had the strongest estimated coefficient in this model. Social influence‘s direct effect was non-significant (*β* = 0.087, *p* = 0.103), yet its indirect effect via perceived enjoyment was significant (*β* = 0.186, *p* < 0.001)—indicating full mediation. Effort expectancy exhibited a marginal direct effect in the conventional MLR estimates (*β* = 0.102, *p* = 0.048), but the cluster-robust estimate was not significant (*β* = 0.098, SE = 0.062, *t* = 1.58, *p* = 0.114). We therefore describe H2 as not supported in the preferred cluster-robust analysis, though the conventional MLR estimate suggested a marginal trend. Perceived risk demonstrated a significant negative effect (*β* = −0.236, *p* < 0.001). Because perceived risk was specified as an observed composite, item-level contribution percentages are not reported.

**Table 5:** Structural model path coefficients, direct and indirect effects.

Path	Original *β* (SE)	Cluster-robust *β* (SE)	*t*-value	Indirect *β*	95% CI	Hypothesis
PE → AI	0.301 (0.048)	0.298 (0.059)	5.05	0.185	[0.128, 0.249]	H1 supported
PE → PEJ → AI	—	—	—	0.185	Same as above	Supplementary mediation
EE → AI	0.102 (0.052)	0.098 (0.062)	1.58	—	—	H2 not supported (cluster-robust)
SI → AI	0.087 (0.053)	0.084 (0.061)	1.38	0.186	[0.131, 0.249]	H3: indirect only
PEJ → AI	0.432 (0.053)	0.425 (0.064)	6.64	—	—	H4 supported
PR → AI	−0.236 (0.041)	−0.231 (0.050)	−4.62	—	—	H5 supported

Model *R*^2^ for AI = 0.61. With prior GenAI experience added as a covariate, all substantive paths remained unchanged in direction and significance; prior experience was not significantly associated with adoption intention (*β* = 0.04, *p* = 0.42). Wald test comparing PEJ and PE coefficients: *χ*^2^ = 4.12, *p* = 0.042.

Bias-corrected bootstrapping (5,000 resamples) revealed a significant indirect effect of performance expectancy on intention through perceived enjoyment [indirect *β* = 0.185, 95% CI = (0.128, 0.249)], confirming partial mediation. Approximately 38% of performance expectancy’s total effect was associated with the enjoyment pathway. Both a-paths were significant (PE → PEJ and SI → PEJ, *p* < 0.001).

### 4.3 Grade-level moderation

Multi-group analysis confirmed positive moderation for the performance expectancy → intention pathway (Table 6). For this pathway, the coefficient for primary students was 0.192 (*p* = 0.004), increasing to 0.376 (*p* < 0.001) for middle-school students, with a critical ratio of −2.43 (*p* = 0.015) and ΔCFI = 0.011—confirming positive moderation.

**Table 6:** Multi-group comparison results by grade level.

Path	Primary *β* (SE)	Middle *β* (SE)	CR Diff	*p*-value	Δ*χ*^2^ (df)	ΔCFI	Conclusion
PE → AI	0.192** (0.066)	0.376*** (0.054)	−2.43	0.015	5.91 (1)*	0.011	Positive moderation
PR → AI	−0.322*** (0.065)	−0.154** (0.056)	2.17	0.030	4.70 (1)*	0.009	Borderline/suggestive
PEJ → AI	0.408*** (0.074)	0.447*** (0.069)	−0.82	0.412	0.67 (1)	0.003	Not significant

*p < 0.05, **p < 0.01, ***p < 0.001. ΔCFI criterion: ≥ 0.01 indicates moderation; ΔCFI = 0.009 for PR → AI falls just below this threshold, so we describe this finding as borderline/suggestive evidence.

For the perceived risk → intention pathway, the primary coefficient was −0.322 (*p* < 0.001), attenuating to −0.154 (*p* = 0.006) in middle school, with CR = 2.17 (*p* = 0.030) and ΔCFI = 0.009. Although ΔCFI fell just below the 0.01 criterion, the significant Satorra–Bentler scaled *χ*^2^ difference test (*p* = 0.030) and the meaningful pattern of coefficient attenuation provide suggestive evidence of moderation. We therefore describe this finding as borderline/suggestive rather than definitive. This pattern suggests that primary students are more sensitive to risk signals—once they perceive potential cognitive harm or information inaccuracy, their adoption intention recedes more sharply. Grade did not significantly moderate the enjoyment–intention link (CR = 0.82, *p* = 0.412, ΔCFI = 0.003), indicating no significant grade difference was detected for this pathway in our sample.

### 4.4 Robustness checks

When re-estimated with cluster-robust standard errors (TYPE = COMPLEX with school as cluster), the same pattern of statistical significance was obtained for all paths except EE → AI. Cluster-robust standard errors were slightly larger than the conventional estimates but yielded the same conclusions for H1, H3, H4, and H5. ICCs for key variables ranged from 0.03 to 0.07, suggesting modest clustering effects.

When prior GenAI experience was added as a control variable, the covariate-adjusted substantive path coefficients remained essentially unchanged. Using cluster-robust standard errors (TYPE = COMPLEX with school as the clustering unit), the estimates were: PE → AI (*β* = 0.298, cluster-robust SE = 0.059, *p* < 0.001), PEJ → AI (*β* = 0.421, cluster-robust SE = 0.065, *p* < 0.001), and PR → AI (*β* = −0.229, cluster-robust SE = 0.051, *p* < 0.001). Prior experience was not significantly associated with adoption intention (*β* = 0.04, *p* = 0.42).

To examine whether the main results differed between prior users (*n* = 480, 64.7%) and non-users (n = 261, 35.3%), we conducted a multi-group analysis comparing these two groups (using cluster-robust SEs). The pattern of path coefficients was largely similar across groups. The only notable difference was that the PE → AI coefficient was somewhat stronger for prior users (*β* = 0.324, SE = 0.064, *p* < 0.001) than for non-users (*β* = 0.258, SE = 0.081, *p* = 0.002), but the difference was not statistically significant (CR = 0.85, *p* = 0.40). These results suggest that the main findings are robust across user and non-user groups.

We acknowledge that the number of school clusters (12) is relatively small for cluster-robust inference; while cluster-robust standard errors with a small number of clusters can be somewhat conservative, the consistency of results across methods supports the robustness of our findings.

## 5 Discussion

### 5.1 Summary of principal findings

This investigation yields four interconnected insights that advance understanding of adolescent technology acceptance in experiential learning contexts.

*First, a pattern in which enjoyment had the largest estimated association with adoption intention was observed among upper-primary and middle-school study-travel participants*. Perceived enjoyment had the largest estimated coefficient in this model and was associated with performance expectancy through an indirect pathway. A Wald test confirmed that the coefficient for PEJ was significantly larger than that for PE (*χ*^2^ = 4.12, *p* = 0.042). This finding diverges from adult work-context findings where usefulness consistently predominates (Venkatesh et al., 2003; Dwivedi et al., 2019), illuminating the substantial psychological reweighting associated with contextual transformation—in study-travel, technology appears to first promise pleasure before it delivers value. This also explains the complete mediation of social influence through enjoyment: peer and teacher endorsements may essentially signal “this is enjoyable” rather than “this is useful.”

*Second, perceived risk constitutes a significant behavioral deterrent*. Students in grades 4–9 prioritized concerns about” information distortion “and” cognitive laziness. “This indicates that even as digital natives, young students harbor a perceptive alertness to technology’s potential to undermine cognitive autonomy, enriching risk perception theory with a developmentally sensitive dimension.

*Third, grade-level moderation reveals systematic differences between primary and middle-school students in this sample*. Primary-stage adoption decisions appear more affect-laden and risk-reactive; middle-school decisions approximate a more instrumentally calibrated pattern. This grade-related difference is consistent with—but not direct evidence of—the emergence of formal operational capacities that enable more systematic evaluation and verification strategies (Inhelder and Piaget, 1958; Crone and Dahl, 2012). We note that grade level may reflect multiple factors including cognitive development, school context, and instruction, and we interpret these observed differences accordingly. The moderation of the risk→intention pathway, however, should be interpreted with caution as the evidence is borderline.

*Fourth, effort expectancy shows a relatively weak incremental association with adoption intention*. The marginal contribution of ease-of-use suggests that after extensive consumer-grade GenAI proliferation, conversational fluency may have become a baseline expectation, no longer constituting a primary driver of adoption. In the preferred cluster-robust analysis, this association was not statistically significant.

### 5.2 Theoretical contributions

Our work advances theory in three directions. *First*, we embed UTAUT2 within the previously unexplored “study-travel × upper-primary and middle-school education” intersection and empirically instantiate an “affective–efficacy” dual-channel model centered on perceived enjoyment—moving beyond static parallel presentations of utilitarian and hedonic motivations to reveal their conditional convergence and pathways. *Second*, the situated recasting of perceived risk and fine-grained moderation analysis push technology threat theory toward more developmentally sensitive considerations, suggesting differentiated risk communication and buffering frameworks calibrated to grade level. *Third*, the full mediation of social influence through enjoyment enriches subjective norm theory by specifying that norm internalization may require positive affective arousal—a boundary condition with novel explanatory power for understanding how social motivations are psychologically transformed in group-based experiential learning contexts.

A potentially fruitful direction for future research is the integration of a distributive justice perspective into technology acceptance models. Although not empirically tested here, recent governance debates on platform fairness raise important questions about how perceptions of institutional equity may moderate AI adoption among young users, especially in educational contexts.

### 5.3 Practical implications

We propose a differentiated framework for study-travel administrators, AI developers, and educators.

*Affective-centric design architecture*. Developers should reconceptualize enjoyment not as superficial embellishment but as core system architecture. Persona-rich, context-adaptive AI agents that dynamically switch roles (e.g., “historical narrator,” “science guide”) based on travel site, coupled with location-triggered narrative generation, puzzle-based inquiry, and creative co-creation modules, can seamlessly integrate useful functions within enjoyable experiences.

*Grade-differentiated functionality and risk-buffering*. For primary students, tools should be configured as “safe play zones” with robust scaffolding, embedded auto-fact-check prompts, and bounded open-domain dialogues. For middle-school students, a “semi-autonomous research mode” featuring multi-source comparison panels, reflection note templates, and AI-confidence scoring can scaffold active questioning and cross-validation.

*Educators as facilitators of “deep play.”* Social influence‘s enjoyment-mediated pathway suggests that teachers’ essential function is not enforcing usage but modeling how to “play deeply.” Pre-trip AI creativity challenges, impromptu inquiry tasks during the journey, and post-trip critical comparison of AI-generated outputs can transform social influence from external regulation into intrinsic enjoyment and intellectual curiosity.

### 5.4 Limitations and future research

Several limitations merit acknowledgment. *First*, the cross-sectional design precludes causal inference; experience sampling methods could capture intra-situational intention dynamics during authentic study-travel. *Second*, the urban sample may not represent rural populations with distinct digital literacy baselines; urban–rural comparative analyses could examine digital divide effects. *Third*, our risk construct was measured with only two items (following the removal of PR3), and did not encompass latent dimensions like algorithmic bias or emotional dependency, nor did it link to objective behavioral data. Future work should develop more comprehensive, age-appropriate risk perception instruments. *Fourth*, GenAI modalities (text-only vs. multimodal, general-purpose vs. education-vertical) may interact with our framework; controlled experimental designs are ongoing. *Fifth*, approximately 35% of participants had heard of GenAI but had not used it prior to the survey; their perceptions of enjoyment, effort expectancy, and performance expectancy may therefore reflect anticipated rather than experienced evaluations. Our sensitivity analysis suggested that the main findings were similar across prior users and non-users, but this limitation should still be considered. Future research with actual user experience or longitudinal designs could address this distinction. *Sixth*, grade level served as a proxy rather than a direct measure of cognitive maturation, and grade is confounded with school type and other contextual factors; the grade-related differences observed should be interpreted as suggestive of developmental processes rather than as definitive evidence of developmental mechanisms, which require direct investigation in future research. *Seventh*, the relatively small number of school clusters (12) in the cluster-robust standard error estimation should be noted; although the consistency of results across methods supports our conclusions, replication with a larger number of schools would be valuable. *Eighth*, the limitations of collecting all variables from the same self-report survey at one time point—including potential common method variance and the inability to establish causal ordering—should be acknowledged. Future research could incorporate objective behavioral data or longitudinal designs to address these concerns.

## Data Availability

The raw data supporting the conclusions of this article will be made available by the authors, without undue reservation.

## References

[ref1] Byrne B. M. Shavelson R. J. Muthén B. (1989). Testing for the equivalence of factor covariance and mean structures: the issue of partial measurement invariance. Psychol. Bull. 105, 456–466. doi: 10.1037/0033-2909.105.3.456

[ref2] Chen F. F. (2007). Sensitivity of goodness of fit indexes to lack of measurement invariance. Struct. Equ. Model. 14, 464–504. doi: 10.1080/10705510701301834

[ref3] Chen X. Zheng Y. (2026). Tourist acceptance of generative AI in cultural heritage interpretation. Tour. Trib. 41, 142–154.

[ref5] Crone E. A. Dahl R. E. (2012). Understanding adolescence as a period of social–affective engagement and goal flexibility. Nat. Rev. Neurosci. 13, 636–650. doi: 10.1038/nrn3313, 22903221

[ref6] Dwivedi Y. K. Rana N. P. Jeyaraj A. Clement M. Williams M. D. (2019). Re-examining the unified theory of acceptance and use of technology (UTAUT): towards a revised theoretical model. Inf. Syst. Front. 21, 719–734. doi: 10.1007/s10796-017-9774-y

[ref7] Featherman M. S. Pavlou P. A. (2003). Predicting e-services adoption: a perceived risk facets perspective. Int. J. Hum.-Comput. Stud. 59, 451–474. doi: 10.1016/s1071-5819(03)00111-3

[ref8] Gupta A. Dwivedi Y. K. Tamilmani K. (2022). Understanding consumer adoption of AI-powered chatbots in retail: a UTAUT2 perspective. J. Bus. Res. 150, 98–110.

[ref9] Huang R. Zhang Y. Li H. (2025). Affective and cognitive determinants of AI companion adoption among adolescents. Comput. Educ. 204:104876.

[ref10] iiMedia Research (2024). 2024–2025 China Study-Travel Economy Market Monitoring Report. Guangzhou, China: iiMedia Research.

[ref11] Inhelder B. Piaget J. (1958). The Growth of logical Thinking from Childhood to Adolescence. New York, United States: Basic Books.

[ref9001] Kline R. B. (2016). Principles and practice of structural equation modeling. (4th ed.). Guilford Press.

[ref13] Li H. Liu Y. Zhang N. (2025). Learning engagement and technology interaction in study-travel contexts: a qualitative investigation. Tour. Forum 18, 55–67.

[ref14] Lin X. Hu A. (2026). Affective energy and social reproduction in experience consumption. Soc. Stud. 41, 180–203.

[ref15] Livingstone S. Stoilova M. (2021). “The 4Cs: classifying online risk to children,” in The SAGE Handbook of child Development and media, (London, United Kingdom: SAGE).

[ref17] Piaget J. (1950). The Psychology of Intelligence. London, United Kingdom: Routledge.

[ref18] Podsakoff P. M. MacKenzie S. B. Lee J. Y. Podsakoff N. P. (2003). Common method biases in behavioral research: a critical review of the literature and recommended remedies. J. Appl. Psychol. 88, 879–903. doi: 10.1037/0021-9010.88.5.879, 14516251

[ref19] Scherer R. Siddiq F. Tondeur J. (2019). The technology acceptance model (TAM): a meta-analytic structural equation modeling approach to explaining teachers’ adoption of digital technology in education. Educ. Technol. Res. Dev. 67, 1047–1071.

[ref20] Schmidt J. Cleveland R. (2023). Developmentally appropriate AI literacy in elementary education. J. Educ. Comput. Res. 61, 1183–1207.

[ref21] Steinberg L. (2005). Cognitive and affective development in adolescence. Trends Cogn. Sci. 9, 69–74. doi: 10.1016/j.tics.2004.12.005, 15668099

[ref22] Tamilmani K. Rana N. P. Dwivedi Y. K. (2021). Consumer acceptance and use of information technology: a meta-analytic review of UTAUT2. Inf. Syst. Front. 23, 987–1005.

[ref23] Venkatesh V. Morris M. G. Davis G. B. Davis F. D. (2003). User acceptance of information technology: toward a unified view. MIS Q. 27, 425–478.

[ref24] Venkatesh V. Thong J. Y. L. Xu X. (2012). Consumer acceptance and use of information technology: extending the unified theory of acceptance and use of technology. MIS Q. 36, 157–178. doi: 10.2307/41410412

[ref26] Wang J. Liu S. Zhang M. (2024). Hedonic experiences and transformative learning in educational tourism. J. Destin. Mark. Manag. 31:100845.

[ref27] Williams L. J. Cote J. A. Buckley M. R. (1989). Lack of method variance in self-reported affect and perceptions at work: reality or artifact? J. Appl. Psychol. 74, 462–468. doi: 10.1037/0021-9010.74.3.462

[ref30] Zhou X. Wang J. (2026). Cognitive diagnosis and tiered cultivation of adolescent AI literacy. E-Educ. Res. 47, 98–107.

